# Maternal separation disrupts noradrenergic control of adult coping behaviors

**DOI:** 10.1038/s41386-025-02201-4

**Published:** 2025-08-28

**Authors:** Chayla R. Vazquez, Léa J. Becker, Chao-Cheng Kuo, Solana A. Cariello, Ayah N. Hamdan, Ream Al-Hasani, Susan E. Maloney, Jordan G. McCall

**Affiliations:** 1 Department of Anesthesiology, Center for Clinical Pharmacology, Washington University Pain Center, Washington University in St. Louis, St. Louis, MO USA; 2 Division of Biology and Biomedical Sciences, Washington University School of Medicine, St. Louis, MO USA; 3 Department of Psychiatry, Washington University School of Medicine, St. Louis, MO USA; 4 Intellectual and Developmental Disabilities Research Center, Washington University School of Medicine, St. Louis, MO USA

**Keywords:** Stress and resilience, Neuronal physiology

## Abstract

Early life stress (ELS) profoundly impacts the brain and correlates with negative affective behaviors in adulthood. The locus coeruleus (LC), a stress-responsive brainstem nucleus that supplies most of the brain with norepinephrine (NE), is known to modulate negative affect. Using repeated maternal separation stress (MSS), we investigated the impact of ELS on the LC and stress-related behaviors in adulthood. We performed ex vivo cell-attached electrophysiology across the lifespan to reveal that MSS significantly increased LC firing during early development and adulthood but not in pre-adolescence and adolescence. We next examined potential changes in the expression of genes linked to LC function. In adulthood, MSS decreased mRNA levels for both the alpha-2_A_ adrenergic receptor and dopamine beta-hydroxylase, the enzyme necessary for NE synthesis. At the behavioral level, MSS increased locomotion in approach-avoidance exploratory assays and increased immobility in the forced swim test. While forced swim increased LC cFos expression, a marker for neuronal excitation, in both No MSS and MSS mice, this increase was significantly lower in MSS mice than in No MSS controls. We further showed that MSS decreased the number of LC cells, possibly underlying the difference in cFos induction and gene expression between MSS and No MSS mice. Finally, we showed that inhibiting the LC in No MSS mice increased immobility time, but did not affect MSS immobility. Instead, LC inhibition in MSS mice increased climbing time. Together, this study demonstrates that MSS dysregulates LC-NE activity across the lifespan and disrupts LC regulation of coping strategies during stressful events.

## Introduction

Early life stress (ELS), including emotional, physical, and/or sexual abuse and neglect, is a major risk factor for poor mental and physical health throughout the lifespan [1–9]. ELS exacerbates stress-related behaviors and susceptibility to negative affective disorders [9–11]. Furthermore, childhood mistreatment profoundly impacts the hypothalamic-pituitary-adrenal axis and stress-related brain regions, such as the central noradrenergic system [8, 12–15]. Recent studies clearly demonstrate the locus coeruleus (LC) – norepinephrine (NE) system plays a critical role in early development and ELS [14, 16–19].

The LC-NE system is a compact brainstem nucleus that extends widespread projections throughout the brain and spinal cord. As the primary NE source in the mammalian forebrain, it plays a key role in negative affective behaviors across species [20–30]. Acute and persistent stressors have long-term effects on LC activity and morphology that increase negative affective behavior in adulthood [31–33]. In adult rodents, acutely enhanced LC activity is necessary and sufficient for stress-induced negative affective behaviors [26, 29, 34–38]. ELS causes substantial, but poorly understood, LC adaptations. For example, sleep deprivation in kittens decreased LC cell size and count [39]. Separately, rats that went through maternal separation stress (MSS) have increased LC cFos, an immediate early gene associated with increased neuronal activity, after re-exposure to stress in adulthood [40]. Additionally, acute MSS changes spontaneous LC firing in rodents, with both increases [17] and decreases being reported [17, 41]. These differences may be due to nuances of the ELS model, species, and/or developmental timepoint. Regardless, ELS likely has a long-term impact on LC activity, but the mechanisms underlying these changes remain largely unknown. Determining the long-term effects of ELS on the LC-NE system is critical for understanding how long-lasting behavioral repercussions are sustained.

Here, we examined the effects of ELS, specifically MSS, on LC neurophysiology throughout the lifetime and adult behavior. We used ex vivo cell-attached electrophysiology and reverse transcription-quantitative polymerase chain reaction (RT-qPCR) to investigate the consequences of MSS on LC function. We found that MSS increased LC activity during early development and adulthood and decreased mRNA expression of important LC-NE genes. Behaviorally, MSS increased immobility in the forced swim test (FST), an assessment of coping behavior. chemogenetic inhibition of the LC during FST in normally-reared mice (No MSS) also increased immobility. While LC inhibition in MSS mice did not change immobility, it increased climbing behavior. Altogether, we show that MSS dysregulates the LC, leading to altered coping behaviors in adulthood.

## Materials and Methods

### Animals

Male and female C57BL/6J (JAX:000664) and Dbh-Cre (JAX:033951) mice were purchased from The Jackson Laboratory (Bar Harbor, ME, USA) and bred in-house. All mice were group-housed, given *ad libitum* access to standard laboratory chow (PicoLab Rodent Diet 20, LabDiet, St. Louis, MO, USA) and water, and maintained on a 12:12-hour light/dark cycle (lights on at 7:00 AM). All experiments and procedures were approved by the Institutional Animal Care and Use Committee of Washington University School of Medicine in accordance with National Institutes of Health guidelines. More husbandry details are available in the Supplementary Materials and Methods.

### Maternal separation stress

Pups were separated from their dam once daily for 4 hours for 7 consecutive days. To do so, the dam was placed in a new separate cage with regular bedding, food, and water for 4 hours during a one-week period (PND 10-16) in a separate room. The pups were left in their home cage on a heating table (~42 °C) to avoid hypothermia. The time of day the maternal separation takes place was varied throughout the week to avoid habituation. The sire was removed prior to PND10.

### Electrophysiology

Acute slices were prepared and recordings were performed as described elsewhere [42, 43] and in the Supplementary Materials and Methods. Cell-attached recordings: Starting at least 48 hours after the final MSS, cells were recorded using the cell-attached recording method in voltage-clamp mode with pipettes filled with recording-aCSF. Whole-cell recordings: For the hM4Di validation experiments, glass pipettes were filled with potassium gluconate-based intra-pipette solution consisting of 120 mM potassium gluconate, 5 mM NaCl, 10 mM HEPES, 1.1 mM EGTA, 15 mM Phosphocreatine, 2 mM ATP and 0.3 mM GTP, pH 7.2–7.3 and osmolality adjusted to 300 mOsm. A green LED light was used to identify viral expression in LC neurons before recording in current clamp mode. To determine the input-output relationship, current injections from -50 to 200 pA with 10 pA steps, were applied while the membrane potential was controlled between -70 to -75 mV. CNO was delivered through the recording-aCSF perfusion system.

### Elevated Plus Maze (EPM)

The EPM is a plus sign-shaped platform elevated off the ground with two enclosed arms and two with open arms. Animals explored the maze for 11 minutes in dim lighting (8–10 lux). The maze was cleaned with 70% ethanol between each trial.

### Open Field Test (OFT)

24 hours after EPM, mice were tested in OFT. OFT testing was performed in a 50 × 50 cm square enclosure for 20 minutes in dim lighting (8-10 lux); the center is defined as a square that is 50% of the total OFT area. The OFT was cleaned with 70% ethanol between each trial.

### Forced Swim Test (FST)

Mice were placed in an opaque 5-liter beaker (30 cm tall x 18 cm in diameter) filled with 3.5 L of 26 ± 1 °C water for a single 6-minute trial. Mice were then towel dried and placed on a heating pad to prevent hypothermia and then returned to their home cage. The last 4 minutes of the trial were analyzed for time spent immobile, the amount of time spent climbing, and the number of climbing attempts (climbing bouts). Climbing was manually counted blindly and defined as the instant when a mouse had both paws touching the side of the container with its back out of the water in an attempt to escape. In Fig. 3, the majority of FST mice previously experienced EPM and OFT one week earlier. Additional mice were added from a second cohort that did not go through EPM or OFT. No differences in distributions were observed between cohorts so both cohorts were merged.

### Immunohistochemistry

Immunohistochemistry was performed as described previously [42, 43] and in the Supplementary Materials and Methods using the following primary and secondary antibodies: Primary: c-Fos (9F6) (rabbit, 1:1000, Cell Signaling Technology, 2250) and tyrosine hydroxylase (TH) (#TYH, 1:1000; Aves Labs Inc., Tigard, OR, USA); Secondary: antibodies Alexa Flour 488 anti-rabbit (1:400; Cat#A-11008; Invitrogen, Carlsbad, California, USA) and Alexa Flour 594 anti-chicken (1:1000; Cat#A-11042; Invitrogen, Carlsbad, California, USA). Images were collected using a Leica confocal microscope (SP8, Leica, Germany). cFos, TH, and DAPI staining were analyzed using ImageJ. To measure both cFos and DAPI, a region-of-interest (ROI) was created around the LC and copied onto the channel that had the cFos or DAPI staining. A threshold for intensity was then created. A binary image of the cFos or DAPI ROI was then made to isolate overlapping signals and count the staining only located within the ROI (Fig. S3). ImageJ was used to count the number of TH-positive cells within an image of the LC (no ROI was used). The intensity feature of ImageJ was also used to measure the intensity of the TH signal.

### Stereotaxic Surgery

Stereotaxic surgery was performed as described [42, 43] and in the Supplementary Materials and Methods. Briefly, a craniotomy was performed, and mice were injected with 350 nL of AAV8-hSyn-DIO-hM4D(Gi)-mCherry (Addgene viral prep # 44362-AAV8) or AAV8-hSyn-DIO-mCherry (Addgene viral prep # 50459-AAV8) bilaterally into the LC (Stereotaxic coordinates from bregma: -5.45 mm anterior-posterior, ± 1.10 mm medial-lateral, and -3.75 mm dorsal-ventral). Both viruses were gifts from Bryan Roth. Mice recovered for at least four weeks prior to behavioral testing and slice electrophysiology.

### RT-qPCR

Tissue was prepared as for electrophysiology and tissue punches were made to isolate individual brain regions as described [43] and in Supplementary Materials and Methods. To generate cDNA, 50 ng of the total mRNA was reversed transcribed with a qScript cDNA synthesis kit (QuantaBio, Beverly, Massachusetts) following manufacturer’s instructions. Real-time quantitative polymerase chain reaction (RT-qPCR) was performed in 10 μL reaction containing 2 μL of cDNA (1/10 dilution), 5 μL of PowerUp SYBR Green Master Mix (Applied Biosystems, Foster City, California, United-States), 2 μL of a mix of forward and reverse primers (10 μM) and 1 μL of H2O. Primer sequences cycling conditions are available in the Supplementary Materials and Methods. Data were normalized to *B2m* (from the same animal), and fold changes were calculated using the 2^-ΔΔCt^ method [44].

### Statistics and data analysis

Statistical significance was defined as *p < 0.05, **p < 0.01, ***p < 0.001, as determined by the Student’s *t*-test (unpaired), Mann-Whitney test, Nested t-test, Two-Way Analysis of Variance (ANOVA), Two-Way Repeated Measures ANOVA followed by Tukey post hoc tests as appropriate, or Kruskal-Wallis for non-normal data with Mann-Whitney pairwise comparisons. In cases where data failed the D’Agostino and Pearson omnibus normality test, non-parametric analyses were used. Statistical analyses were performed in GraphPad Prism 10.

## Results

### Maternal separation increases locus coeruleus firing during early development and adulthood

**Fig. 1:**
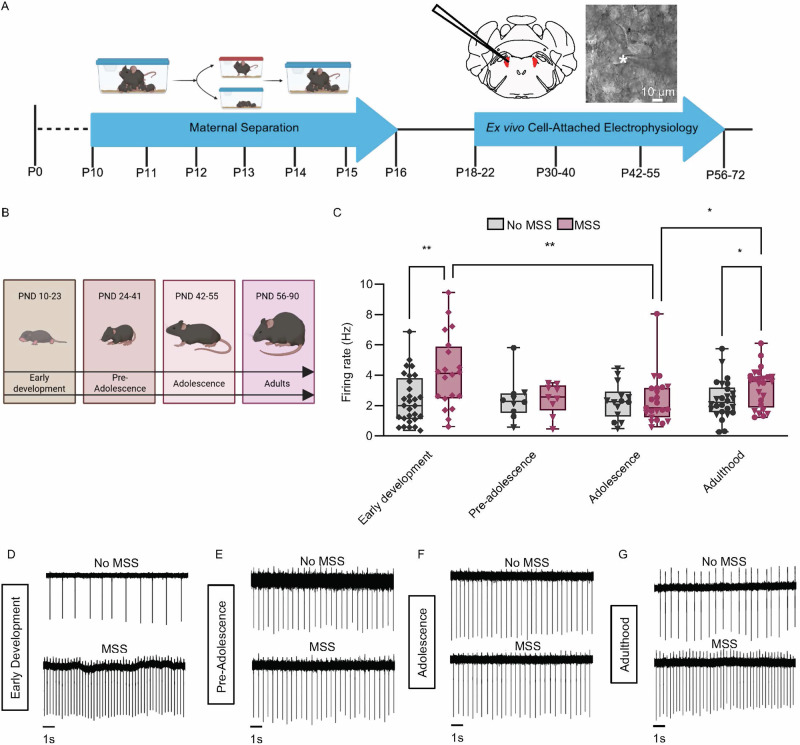
Maternal separation increases LC activity during early development and adulthood. **A** Timeline showing the maternal separation paradigm and electrophysiological time points, including a visual representation of the LC and electrode with a DIC image of a recorded LC neuron (*marks the pipette tip). **B** A diagram defining different mouse developmental time points during specific age ranges. **C** Firing rate of No MSS and MSS groups at all four life stages. Early development: No MSS (*n* = 29 cells from 5 mice) and MSS (*n* = 20 cells from 5 mice). Pre-adolescence: No MSS (*n* = 10 cells from 2 mice) and MSS (*n* = 9 cells from 2 mice). Adolescence: No MSS (*n* = 14 cells from 4 mice) and MSS (*n* = 23 cells from 5 mice). Adulthood: No MSS (*n* = 26 cells from 5 mice) and MSS (*n* = 25 from 6 mice). Non-parametric analysis, Kruskal-Wallis for non-normal data with Mann-Whitney pairwise comparisons. KW: Group, X²(7,*N* = 156) = 18.874, *p* = .009 (Group variable has 8 levels, MSS and No MSS at each age. Data are presented as a box and whiskers plot, where the box represents the interquartile range (IQR), the line within the box indicates the median, and whiskers extend from the minimum to maximum values. Representative LC recordings from No MSS mice (top) and MSS mice (bottom) during early development (**D**), pre-adolescence (**E**), adolescence (**F**), and adulthood (**G**). Triangles ▼ in graphs indicate male animals; circles ● indicate female animals; ♦ indicate sex not recorded. Sex was not recorded for pups.

Prior work in rats has shown that MSS combined with experimenter handling altered LC activity 1-3 weeks later [17]. Importantly, however, the changes in LC activity varied depending on handling duration, with short handling (15 minutes) decreasing LC spontaneous firing rate and longer handling (180 minutes) increasing LC spontaneous firing rate [17]. Therefore, we sought to investigate the effects of MSS alone throughout the lifespan to determine when MSS-induced LC firing rate changes occur and how long these changes last. For our MSS paradigm, mice experienced 4 hours of maternal separation from postnatal days (PND) 10 to 16, similar to previous work but with sufficient nesting material [45, 46]. We maintained normally-reared control mice (No MSS) in their normal homecage breeding environment. We then performed ex vivo electrophysiological recordings from early development to adulthood (ages established through previous research [47]) (Fig. 1A–C). In early development, MSS significantly increased spontaneous LC firing (Fig. 1D). This effect transiently disappeared during pre-adolescence and adolescence (Fig. 1E, F) and returned in adulthood (Fig. 1G). Together, MSS increased spontaneous LC activity shortly after MSS and drove a similar increase in adulthood.

#### Maternal separation decreases adult expression of genes associated with LC-NE function

**Fig. 2:**
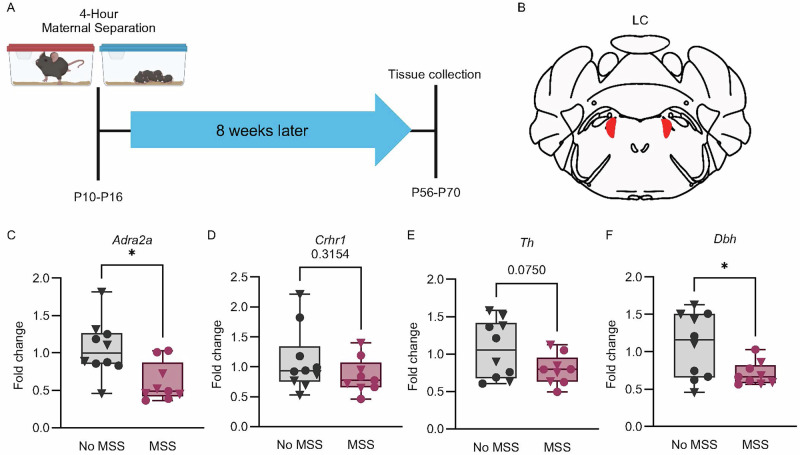
MSS alters mRNA content of the LC. **A** Timeline showing the time of tissue collection for the mRNA extraction after MSS. Tissue from No MSS mice was collected within the same age range. **B** Schematic of LC tissue collection. **C** *Adra2a* expression in the LC was significantly decreased in MSS animals compared to No MSS animals (Mann-Whitney test, U = 14, *p* = 0.0101). **D** *Crhr1* expression remained unaltered by MSS (Mann-Whitney test, U = 32, *p* = 0.3154) (**E**) *Dbh* expression in the LC was significantly decreased in the MSS animals compared to No MSS animals (Student’s *t*-test, t(17) = 2.503, *p* = 0.0228). **F** There was no difference in *Th* or expression in the LC between MSS and No MSS mice (Student’s *t*-test, t(17) = 1.897, *p* = 0.0750). No MSS (*n* = 10 mice) and MSS (*n* = 9 mice). Triangles ▼ in graphs indicate male animals; circles ● indicate female animals. Data are presented as box and whiskers plots, where the box represents the IQR, the line within the box indicates the median, and whiskers extend from the minimum to maximum values.

Several mechanisms regulate LC activity. Notably, norepinephrine release exerts autoinhibitory feedback by activating to alpha-2_A_ adrenergic receptors (ADRA2A) [48, 49]. Prior work suggests that ELS modulates ADRA2A function in humans and animal models [50–52]. Stress also recruits the corticotropin-releasing hormone (CRH) system, and extensive evidence demonstrates that CRH increases LC activity through binding to the corticotropin-releasing hormone receptor 1 (CRHR1) [26, 53–58]. Therefore, we measured the expression of *Adra2a* and *Crhr1* mRNA in the LC region from adult MSS and No MSS mice using RT-qPCR (Fig. 2A&B). We found MSS significantly decreased *Adra2a* but not *Crhr1* expression (Fig. 2C&D), suggesting that reduced *Adra2a* expression in the LC may contribute to the increase in LC firing observed in adulthood after MSS. Dopamine beta-hydroxylase (DBH) and tyrosine hydroxylase (TH) are the two main enzymes in the NE biosynthesis pathway [59]. TH, the rate-limiting enzyme for catecholamine synthesis, converts tyrosine into L-DOPA, which is then converted into dopamine [60, 61]. DBH acts downstream to convert dopamine into norepinephrine [61]. Both enzymes determine the availability of dopamine as a substrate for norepinephrine production. Therefore, to determine if the increased firing activity of the LC was accompanied by enhanced NE synthesis, we measured the expression of *Th* and *Dbh* in MSS and No MSS mice. Surprisingly we found that MSS decreased *Dbh* expression in the LC region, which may indicate a compensatory mechanism in response to the increased LC activity. No significant differences were found in *Th* expression (Fig. 2E&F). Finally, to identify potential downstream effects, we examined adrenergic receptor expression in brain regions relevant for stress-related behaviors that receive input from the LC including the basolateral amygdala, the central amygdala, and the anterior cingulate cortex [62–69]. Here we saw no significant changes in downstream adrenergic receptor mRNA expression (Figure S1).

#### Maternal separation increases adult passive coping in the forced swim test

**Fig. 3:**
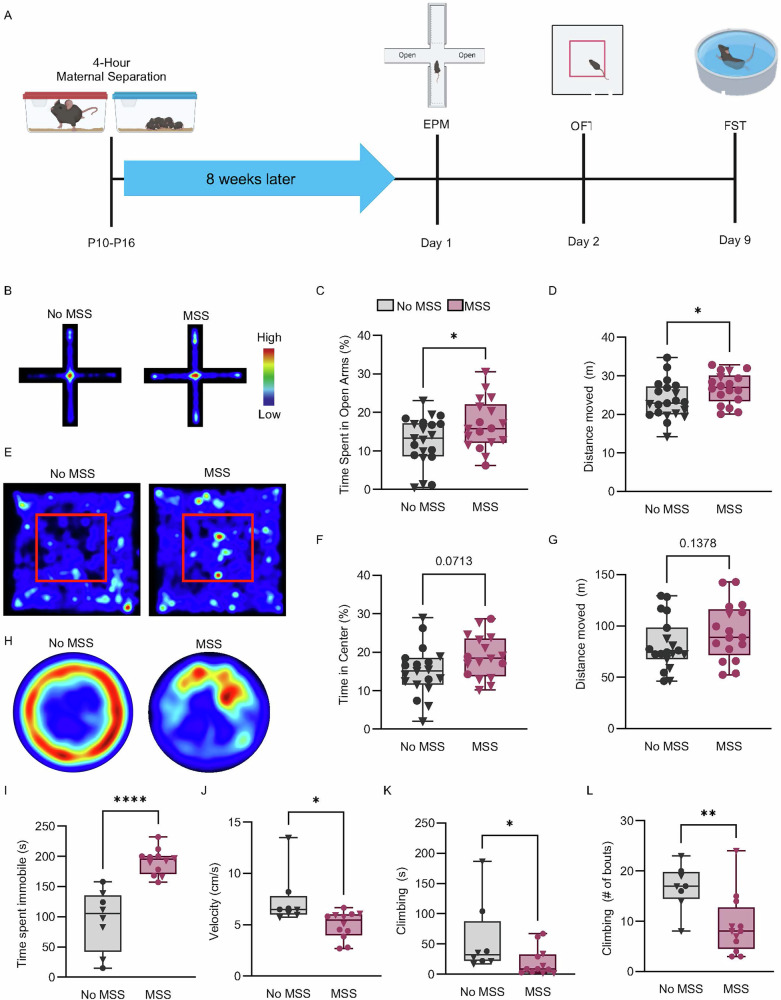
Maternal separation increases passive coping behavior during Forced Swim Test. **A** Timeline showing the timing of behavioral tests 8 weeks after maternal separation. **B** Representative heat maps showing activity during the elevated plus maze. **C** The percentage of time spent in the open arms of the elevated plus maze (No MSS (*n* = 21) and MSS (*n* = 18), unpaired t-test, t(37) = 2.098, *p* = 0.0428). **D** The total amount of distance moved in the elevated plus maze (Unpaired t-test, t(37) = 2.171, *p* = 0.0364). (**E**) Representative heat maps showing activity during the open field test. **F** The percentage of time spent in the center of the open field test (No MSS (*n* = 19) and MSS (n = 17), Unpaired t-test, t(34) = 1.861). **G** The total distance moved in the open field test (Mann-Whitney, U = 114). **H** Representative heat maps showing activity during the forced swim test. (**I**) The amount of time spent immobile during the forced swim test for the No MSS and MSS group (No MSS (*n* = 8) and MSS (n = 12), Unpaired t-test, t(18) = 5.924, ****p < 0.0001). **J** The average velocity during the forced swim test for the No MSS and MSS group (Unpaired t-test, t(18) = 2.760, *p* = 0.0129). **K** The amount of time spent climbing during the forced swim test for the No MSS and MSS group (Mann-Whitney, U = 20, *p* = 0.0296). **L** The number of climbing bouts during the forced swim test for the No MSS and MSS group (Unpaired t-test, t(18) = 3.041, *p* = 0.0070). Triangles ▼ in graphs indicate male animals; circles ● indicate female animals. Data are presented as box and whiskers plots, where the box represents the IQR, the line within the box indicates the median, and whiskers extend from the minimum to maximum values.

After identifying increased LC spontaneous firing rate and downregulated *Adra2a* and *Dbh* expression in the LC in adult mice after MSS, we then tested whether these changes aligned with negative affective behaviors associated with LC function. We used three different stress-related behavior tests: the elevated plus maze (EPM), open field test (OFT), and forced swim test (FST) (Fig. 3A). Similar to previous studies [70, 71], MSS mice spent more time in the open arms of the EPM compared to No MSS mice (Fig. 3B, C). Additionally, MSS increased locomotion compared to No MSS mice (Fig. 3D). Both of these effects appear to be largely driven by male MSS mice, but these results are likely underpowered to determine a true sex difference (Figure S2A, B). We found no changes in time spent in the center or the distance moved in the OFT between MSS and No MSS (Fig. 3E–G). However, we again observed that MSS males had increased total distance traveled compared to No MSS males in OFT (Fig. S2D). Finally, in the FST, which measures coping behavior during a stress-inducing experience [72–76], MSS significantly increased immobility and decreased velocity (Fig. 3H–J). Furthermore, MSS significantly reduced time spent climbing and the number of climbing bouts during FST compared to No MSS controls (Fig. 3K, L). Together these results indicate MSS increased passive coping behavior in adult mice. Importantly, FST was the only assay where MSS mice significantly differed from No MSS mice in both sexes and FST is modulated by noradrenergic activity [37], thus making it the most selective indicator for MSS-altered, potentially LC-related negative affective behavior. Because the FST is likely the most stressful test we used, our results could further indicate altered stress responsivity in MSS animals.

#### Forced swim test activates LC neurons and maternal separation decreases LC size and cell count

**Fig. 4:**
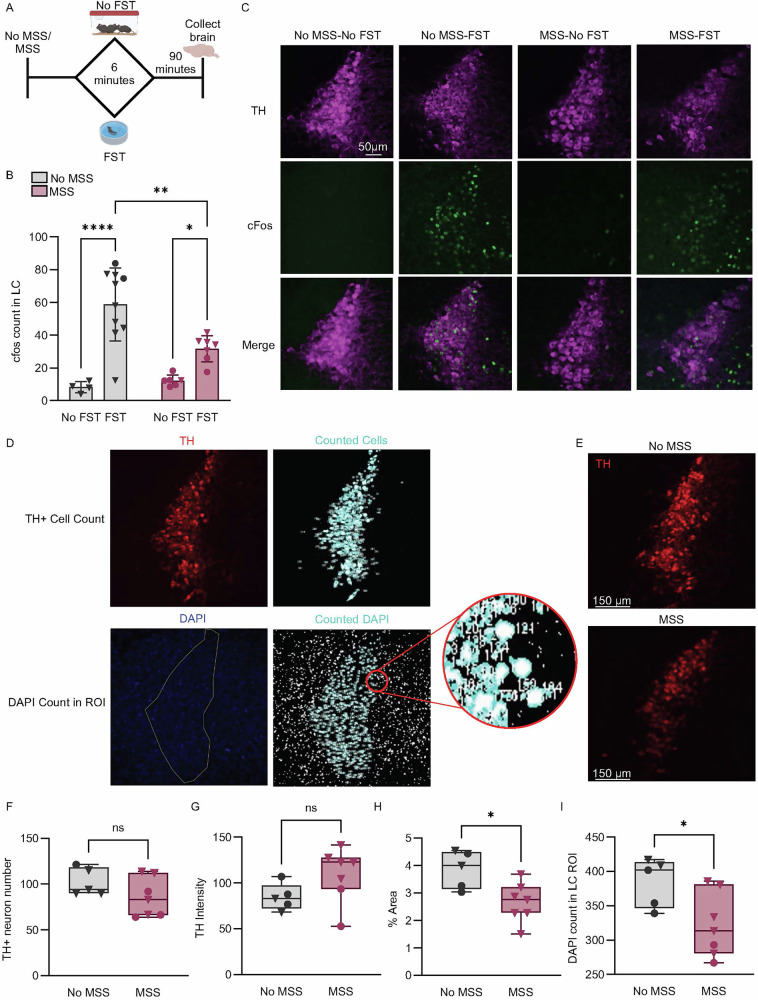
Forced Swim Test increases LC cFos expression. **A** Timeline for quantification of cFos expression within LC after the forced swim test. The FST mice went through FST for 6 minutes and were sacrificed 90 minutes afterward. The No FST mice did not go through FST and instead sat outside of their colony room for 6 minutes. After 90 minutes they were sacrificed. **B** The mean cFos count in the LC per animal within each group (*n* = 4-10 mice/group. The mean from 3-5 slices per animal was plotted. Data represented as mean ± SEM, Two-way ANOVA test, F(1, 24) = 7.287, ****p < 0.0001, ***p = 0.0006, *p = 0.0348). **C** Confocal images of the cFos. **D** Images counting both TH+ cells and DAPI within a TH-defined ROI. **E** Confocal images of TH+ neurons in the LC from No MSS and MSS mice. **F** TH+ neurons in the No MSS and MSS group (Nested t-test, t(10) = 1.896, *p* = 0.0872). **G** Intensity of TH+ staining (a.u.) in each LC slice image in the No MSS and MSS group (Nested t-test, t(10) = 0.2634, *p* = 0.7976). **H** % area of TH+ staining within the entire image in the No MSS and MSS group (Nested t-test, t(10) = 2.932, *p* = 0.0152). **I** DAPI count within a TH-defined ROI in the No MSS and MSS group (Nested t-test, t(10) = 2.966, *p* = 0.0141). Data (**F**–**I**) are presented as box and whiskers plots, where the box represents the IQR, the line within the box indicates the median, and whiskers extend from the minimum to maximum values. *n* = 5–7 mice/group, data nested by slice. Triangles ▼ in graphs indicate male animals; circles ● indicate female animals.

To determine if MSS disrupts LC response to stress in adulthood, we tested whether FST differentially activates the LC across groups. We measured the expression of LC cFos, a marker of neuronal activation [77], either following FST or no manipulation in control and MSS mice (Fig. 4A). We counted cFos within an anatomically defined TH-defined ROI that encapsulated the LC (Fig. S3). FST significantly increased cFos in the LC ROI in both No MSS and MSS groups (Fig. 4B, C). However, FST-induced cFos expression was significantly lower in MSS mice compared to No MSS controls. One explanation for the decreased LC cFos+ cells could be a decrease in total LC cells following MSS. We therefore measured the number of TH+ neurons, TH+ intensity, the area of TH+ expression, and the amount of DAPI within a ROI outlining the LC in adult mice with and without MSS (Fig. 4D, E). MSS did not significantly alter the number of TH+ neurons or the intensity of TH staining (Fig. 4F, G). However, MSS decreased total TH area and the amount of DAPI within the LC ROI (Fig. 4H, I). Thus, LC is recruited and active during FST, but MSS-induced alterations to the LC may prevent an optimal LC response to forced swim and other behavioral challenges.

#### Decreasing LC excitability increases FST climbing in MSS mice

**Fig. 5:**
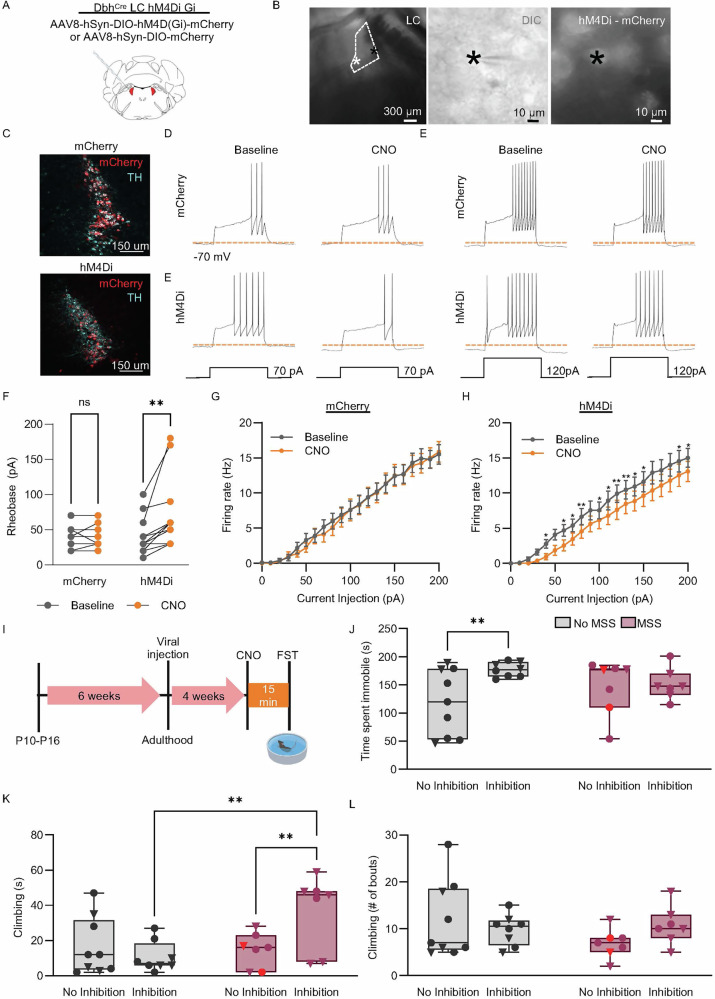
LC inhibition differentially alters coping behaviors in No MSS and MSS mice. **A** Diagram of LC targeting strategy. **B** Left, a DIC image under low magnification of the LC with the two recording pipettes marked by asterisks. Middle, an image for one of the recorded cells shown in the left panel is indicated by a black asterisk. Right, a fluorescent image of the same recorded LC neuron showing hM4Di-mCherry expression. **C** Confocal image showing the colocalization of mCherry or hM4Di-mCherry with *TH* immunoreactive signals. Representative traces from an mCherry and hM4Di-mCherry expressing LC neurons at baseline and with CNO administration upon current injection at 60 (**D**) and 100 (**E**) pA. (**F**) Rheobase in mCherry (*n* = 10) and hM4Di-mCherry (*n* = 13) expressing LC neurons at baseline and under CNO administration (Two-way ANOVA, F(1, 27) = 4.343, **p = 0.0013). Plots demonstrating input-output relationship from current injections in mCherry (**G**) and hM4Di-mCherry (**H**) expressing LC neurons at baseline and after CNO is administered (G: Two-way ANOVA, F (1, 16) = 0.03017; **H**: Two-way ANOVA, F(1, 24) = 1.580, *p < 0.05 and **p < 0.01). **I** In vivo experimental timeline. **J** Immobility in forced swim test from No MSS and MSS animals, all animals expressing hM4Di-mCherry received either saline or CNO prior to the test except two animals MSS no inhibition animals that express mCherry and received CNO (denoted in red) (No MSS^No LC Inhibition^ (*n* = 9); No MSS^LC Inhibition^ (*n* = 8); MSS^No LC Inhibition^ (*n* = 7); MSS^LC Inhibition^ (*n* = 7); Two-way ANOVA with Uncorrected Fisher’s LSD, F (1, 27) = 4.343, *p* = 0.0468). **K** Time spent climbing in the forced swim test in the No MSS and MSS group (Non-parametric analysis, Kruskal-Wallis (KW) for non-normal data with Mann-Whitney pairwise comparisons. KW: Group, X²(3, *N* = 31) = 8.342, *p* = .039 (Group variable has 4 levels, MSS and No MSS for inhibition & No inhibition). **L** Number of climbing bouts during the forced swim test in the No MSS and MSS group (Non-parametric analysis, Kruskal-Wallis (KW) for non-normal data with Mann-Whitney pairwise comparisons. KW: Group, X²(3, *N* = 31) = 1.188, *p* = .756 (Group variable has 4 levels, MSS and No MSS for inhibition & No inhibition).

After observing that the LC is recruited during FST and that LC baseline firing rate is significantly increased after MSS, we sought to reverse the effects of MSS-induced immobility during FST by inhibiting the LC. To do so, we injected a virus to selectively express either inhibitory DREADD receptors (designer receptors exclusively activated by designer drugs; hM4Di; AAV8-hSyn-DIO-hM4D(Gi)-mCherry) or mCherry alone (AAV8-hSyn-DIO-mCherry) in the LC of *Dbh*^Cre^ mice (Fig. 5A). We used whole-cell current clamp to functionally validate hM4Di expression (Fig. 5B–H). Here, bath application of CNO significantly increased the current needed to elicit an action potential (i.e., the rheobase) in hM4Di+ LC neurons, but not in mCherry+ LC neurons (Fig. 5C–F). Furthermore, in response to increasing current, the firing rate for mCherry did not change with CNO application (Fig. 5G). However, CNO application on hM4Di-expressing neurons shifted firing rate significantly rightward (Fig. 5H). Together, this increased rheobase and decreased firing in response to current suggests that CNO-mediated activation of hM4Di decreased LC excitability. We next tested whether blunting LC excitability in MSS animals could rescue MSS-induced immobility during FST (Fig. 5I, J). Here we administered CNO (3 mg/kg, i.p.) or saline 15 minutes prior to FST in both No MSS and MSS mice expressing hM4Di (Fig. 5I) (NB: two MSS mice instead expressed mCherry and received saline to serve as No Inhibition controls). In No MSS mice, LC inhibition (No MSS^LC Inhibition^) increased immobility. In MSS mice, however, LC inhibition had no effect on immobility in MSS mice (MSS^LC Inhibition^) (Fig. 5J). Interestingly, MSS^LC Inhibition^ had increased climbing compared to No MSS^No LC Inhibition^ and No MSS^LC Inhibition^ (Fig. 5K). There was, however, no change in the number of attempted climbs between any groups (Fig. 5L). This suggests that MSS differentially alters the LC’s ability to regulate passive coping strategies.

## Discussion

Literature on stress-related and coping behaviors after MSS is inconsistent. One possibility for the discrepancies across studies is the developmental timepoint(s) at which MSS occurred. It is not yet clear how these differences could impact LC activity and behavior. For instance, MSS from PND 2-12 in a strain of C57BL/6 mice, earlier than our manipulation from PND10-16 had no effect in the time spent in the open arms of the EPM or the time spent in the center area of the OFT during adolescence or adulthood [78]. However, this study, like ours, identified increased immobility during the FST in adolescence. We did not test behavior during adolescence, but observed this effect in adulthood. In another C57BL/6 mouse, MSS from PND 2-14 decreased open arm time in the EPM for MSS females compared to No MSS mice [79]. Additionally, MSS males decreased locomotor activity in the OFT compared to No MSS mice – the opposite effect we observed. Other work showed C57BL/6J pups that experienced MSS during the late postnatal period (PND 10-20, running later than our MSS) and then experienced social defeat had decreased social interaction, decreased sucrose preference, and increased immobility during FST compared to pups that had MSS during the early postnatal period (PND 2-12) which were proportionate to No MSS mice [45]. More recently, a similar MSS timeline (PND6-16) combined with early weaning and adolescent stress showed increased locomotion during EPM, but decreased time spent in open arms in female MSS BALB/cJ mice [41]. Remarkably, this study showed decreased LC excitability following these early and adolescent stressors. Although our data in C57BL/6J mice align with earlier research showing an increase in LC activity after MSS [17], a follow-up study from the same group using C57BL/6J mice showed similar decreases as well as sex differences arising from dimorphic responses to CRH [80, 81]. Together, these apparently conflicting reports suggest that LC adaptation to MSS is dependent on the timepoint and duration of MSS, the length of each separation, and the inclusion of other stressors. At the behavioral level, however, one of the most consistent results across strains and timepoints is that in the absence of an acute stressor, locomotor activity is often increased (as we see in Fig. 2D) [41, 79, 82, 83]. Sometimes, this effect drives increased exploratory behavior, often interpreted as anxiolytic (Fig. 2C). Importantly, however, when challenged with an acute stressor such as the FST, negative affective behaviors more reliably emerge (Fig. 2I) [82–85]. Furthermore, ultimate understanding is likely more complex than a binary increase or decrease in tonic firing, consistent with our evolving understanding of LC firing across strains, species, and timepoints [86]. The LC undergoes substantial postnatal development [87]. In early life, the LC has a broad spontaneous firing range and gap junction-mediated electrotonic-coupling between neurons that is greatly reduced over time [88–90]. These factors play a key role in physiological changes observed in the LC during early development. Behaviorally, the LC responds strongly to both noxious and innocuous stimuli in early development, but responsiveness shifts towards noxious stimuli as the animal ages [87]. Detailed mechanisms of the long and intricate process of LC maturation following birth will be critical to understand the consequences of disrupting LC activity during early development.

ELS has been shown to modify the transcriptomic profile across many brains regions [45, 91–94]. In our study, we focused on a few genes that are either known to regulate LC activity or related to NE synthesis. For instance, *Crh*, which is implicated in stress response and known to increase LC activity, is upregulated in the hypothalamus of animals that went through MSS from PND 5 to PND 21 for 6 hours each day [95], which could have functional consequences for the LC and its associated circuitry. While *Crhr1* mRNA was not changed in our paradigm, this does not rule out the possibility of receptor desensitization or a change in CRH release in the LC. We did find that MSS decreased *Adra2a* mRNA in LC, which could contribute to increased LC activity following MSS. We further observed decreased *Dbh* expression that may reflect a compensatory downregulation of enzymatic activity to counteract LC hyperactivity. Interestingly, we did not detect any changes in *Th* expression, raising the possibility of increased levels of dopamine. Further research measuring dopamine and NE levels in the LC and its output regions would give valuable insight into the effect of MSS on the LC-NE system. Importantly, our MSS paradigm reduced the number of cell nuclei within the LC of adult mice. Of note, a similar decrease in LC cell count was also reported in sleep-deprived kittens [39] and altered morphology and decreased cell counts have been found in cell types other than brain regions of rats and mice following various forms of ELS [96–99]. Since our gene expression analysis was done on tissue dissection of the whole LC region, we cannot rule out that this contributes to the decrease in *Dbh* and *Adra2a* expression. However, we would also expect a decrease in *Th* and *Crhr1*, which we did not observe. Finally, our study only focused on gene expression in adulthood. Performing similar experiments across the lifespan could highlight possible differences or similarities in the mechanisms leading to LC hyperactivity in early development and adulthood and give insight into why we did not observe such effects in pre-adolescence and adolescence.

The central noradrenergic system is known to modulate coping behaviors in FST. Norepinephrine infusion into the LC of naïve rats non-linearly affects immobility with doses differentially increasing and decreasing FST immobility [36]. This alpha2a-mediated immobility during forced swim is driven by alpha-2_A_ receptors on non-LC cells [100]. Furthermore, inhibiting TH by a-methyl-para-tyrosine methyl ester (AMPT) increases immobility for mice that have transgenic, lifelong corticotropin-releasing hormone (CRH)-mediated LC hyperactivation [55]. These latter results align with our study showing chronically enhanced LC activity and downregulated *Dbh* expression. Finally, antidepressants that increase extracellular norepinephrine increase climbing behaviors during active coping [101, 102]. To determine the functional relevance of increased LC activity, we decreased LC excitability following MSS. Inhibiting the LC in No MSS animals increased immobility similar in scale to how MSS increased immobility. While these results appear at odds (i.e., hM4Di decreases LC excitability and MSS increases LC firing), both phenomena push the LC activity outside of the optimal firing rate range into hypo- and hyper-firing rate ranges, respectively. This appears to be a possible example of the Yerkes-Dodson law where both extremes lead to dysfunctional behavior [103–108]. Interestingly, however, we did not see decreased immobility in MSS mice, but we did see increased time spent climbing in these animals.

This result also appears in contrast to what would be expected (i.e., norepinephrine reuptake inhibitors increase climbing), further suggesting that MSS induces long-term adaptations in the noradrenergic system that inhibit the ability of the LC to modulate behavior normally. It is also possible that the DREADD-mediated decreased excitability we observed in the slice is overwhelmed by endogenous synaptic input in vivo in MSS mice. Likewise, adaptations in intracellular signaling could be disrupted after MSS. Either case could render the chemogenetic manipulation less functional, and future studies should more closely examine both the intracellular and brain-wide impact of chronically increased LC firing. Many studies have investigated the effects of manipulating LC tonic and phasic activation on different behaviors through optogenetic activation [26, 109–111]. However, few have tested manipulating LC activity after it is naturally firing at a suboptimal rate to see changes in behavior. Altogether, this study provides insight into how dysregulated LC activity after MSS could prevent the LC from optimally controlling behavior.

## Supplementary information

Supplemental Methods and Figures

Dataset 1

## Data Availability

All data presented in this manuscript is available in Dataset 1.
