# Supplementary material for: Maternal separation disrupts noradrenergic control of adult coping behaviors
Source: Neuropsychopharmacology. 2025 Aug 28;50(12):1795–806. doi: 10.1038/s41386-025-02201-4 (PMC12518818; doi:10.1038/s41386-025-02201-4)
Supplement: Supplementary file 1 — Supplemental Methods and Figures [file 41386_2025_2201_MOESM1_ESM.docx]

**Supplementary Information for:**

**Maternal separation disrupts noradrenergic control of adult coping behaviors**

Chayla R. Vazquez^1,2^, Léa J. Becker^1^, Chao-Cheng Kuo^1^, Solana A. Cariello^1^, Ayah N. Hamdan^1^, Ream Al-Hasani^1,2^, Susan E. Maloney^3^, Jordan G. McCall^1,2*^

^1^Department of Anesthesiology, Center for Clinical Pharmacology, Washington University Pain Center, Washington University in St. Louis, St. Louis, MO, USA.

^2^Division of Biology and Biomedical Sciences, Washington University School of Medicine, St. Louis, MO, USA.

^3^Department of Psychiatry, Washington University School of Medicine, St. Louis, MO, USA; Intellectual and Developmental Disabilities Research Center, Washington University School of Medicine, St. Louis, MO, USA

*To whom correspondence should be addressed:

J.G.M.: Address: 660 S. Euclid Ave, Box 8054, St. Louis, MO, USA, 63110

E-mail: jordangmccall@wustl.edu Website: www.mccall-lab.org.

**Supplementary Materials and Methods**

**Figures S1-S3**

**Supplementary Materials and Methods:**

Animals

Male and female C57BL/6J (JAX:000664) and Dbh-Cre (JAX:033951) mice were purchased from The Jackson Laboratory (Bar Harbor, ME, USA) and bred in-house. Adult animals were either transferred to a holding facility adjacent to the behavioral space between 4-6 weeks of age or bred directly in the holding facility. Pups used in the experiments were only bred in the holding facility. All mice were group-housed, given *ad libitum* access to standard laboratory chow (PicoLab Rodent Diet 20, LabDiet, St. Louis, MO, USA) and water, and maintained on a 12:12-hour light/dark cycle (lights on at 7:00 AM). Prior to all behavioral experiments, mice were habituated in the room where the experiment was taking place for 30 minutes before the start of the experiment. All experiments and procedures were approved by the Institutional Animal Care and Use Committee of Washington University School of Medicine in accordance with National Institutes of Health guidelines.

Acute Slice Preparation

Adult mice were deeply anesthetized via a i.p. injection of a mix of ketamine (69.57 mg/ml), xylazine (4.35 mg/ml), & acepromazine (0.87 mg/ml). Upon sedation, adult mice were perfused with slicing-aCSF consisting of 92 mM N-methyl-d-glucose (NMDG), 2.5 mM KCl, 1.25 mM NaH_2_PO_4_, 10 mM MgSO_4_, 20 mM HEPES, 30 mM NaHCO_3_, 25 mM glucose, 0.5 mM CaCl_2_, 5 mM sodium ascorbate and 3 mM sodium pyruvate, oxygenated with 95% O_2_ and 5% CO_2_. pH of aCSF solution was 7.3–7.4. Pups were anesthetized via exposure to isoflurane that was dropped on tissue in a mouse transfer container. Upon sedation, pups were decapitated, and the cranium was submerged in aCSF immediately after. In both adults and pups, the brain was dissected and embedded with 2% agarose in slice-aCSF. Coronal brain slices were cut into 300 μm slices using a vibratome (VF310-0Z, Precisionary Instruments, MA, USA) and incubated in warm (32°C) slicing-aCSF for 10 mins. After incubation slices were transferred to holding-aCSF containing 92 mM NaCl, 2.5 mM KCl, 1.25 mM NaH_2_PO4, 30 mM NaHCO_3_, 20 mM HEPES, 25 mM glucose, 2 mM MgSO_4_, 2 mM CaCl_2_, 5 mM sodium ascorbate and 3 mM sodium pyruvate, oxygenated with 95% O_2_ and 5% CO_2_ for one hour. pH of the solution was 7.3–7.4. Slices were placed into a recording chamber mounted on an upright microscope (BX51WI, Olympus Optical Co., Ltd, Tokyo, Japan) with epifluorescence equipment and a highspeed camera (ORCA-Flash4.0LT, Hamamatsu Photonics, Shizuoka, Japan) while perfused continuously with warm (29–31°C) recording-aCSF containing 124 mM NaCl, 2.5 mM KCl, 1.25 mM NaH_2_PO_4_, 24 mM NaHCO_3_, 5 mM HEPES, 12.5 mM glucose, 2 mM MgCl_2_, 2 mM CaCl_2_, oxygenated with 95% O_2_ and 5% CO_2_ and pH 7.3–7.4.

Electrophysiology

All recordings were performed using visual guidance (40× water immersion objective lens, LUMPLFLN-40xW, Olympus, Tokyo, Japan) through a glass pipette pulled from borosilicate glass capillary (GC150F-10, Warner Instruments, Hamden, CT, USA) with a resistance from 2-5 MΩ. All data were collected using a Multiclamp 700B amplifier (Molecular Devices, San Jose, CA, USA) low-pass filtered at 2 kHz and digitized at 10 kHz through Axon Digidata 1440A interface (Molecular Devices, CA, USA) running Clampex software (Molecular Devices, CA, USA). Data was exported through Clampex software and analyzed using Easy Electrophysiology (Easy Electrophysiology Ltd, London, England) and GraphPad Prism 10 (GraphPad Software, MA, USA). Cell-attached recordings: Starting at least 48 hours after the final MSS, cells were recorded using the cell-attached recording method in voltage-clamp mode with pipettes filled with recording-aCSF. Whole-cell recordings: For the hM4Di validation experiments, glass pipettes were filled with potassium gluconate-based intra-pipette solution consisting of 120 mM potassium gluconate, 5 mM NaCl, 10 mM HEPES, 1.1 mM EGTA, 15 mM Phosphocreatine, 2 mM ATP and 0.3 mM GTP, pH 7.2–7.3 and osmolality adjusted to 300 mOsm. A green LED light was used to identify viral expression in LC neurons before recording in current clamp mode. To determine the input-output relationship, current injections from -50 to 200 pA with 10 pA steps, were applied while the membrane potential was controlled between -70 to -75 mV. CNO was delivered through the recording-aCSF perfusion system.

Tissue collection for qPCR

*Locus coeruleus.* Mice were anesthetized with a mix (i.p. 182 mg/kg) of ketamine (69.57 mg/ml), xylazine (4.35 mg/ml), & acepromazine (0.87 mg/ml) and perfused with ice-cold aCSF containing the following (in mM): 92 N-methyl-d-glucose (NMDG), 2.5 KCl, 1.25 NaH_2_PO_4_, 10 MgSO_4_, 20 HEPES, 30 NaHCO_3_, 25 glucose, 0.5 CaCl_2_, 5 sodium ascorbate and 3 sodium pyruvate, oxygenated with 95% O_2_ and 5% CO_2_. pH of aCSF solution was 7.3–7.4 and osmolality adjusted to 315–320 mOsm with sucrose. The brainstem was dissected and embedded with 2% agarose in aCSF and coronal brain slices were cut into 250 μm slices using a vibratome (VF310-0Z, Precisionary Instruments, MA, USA). Bilateral LC was dissected under a microscope (Leica S6E, Leica Microsystem GmbH), immediately frozen, and kept at -80 °C until RNA extraction.

*Other tissues.* Following brainstem isolation, the remaining rostral part of the brain was cut into 1mm thick coronal slices using a brain matrix (Zivic Instruments). Slices containing the ACC, CeA, and BLA were transferred in a petri dish filled with aCSF and put under a microscope. Bilateral ACC were dissected using anatomical landmarks. Bilateral CeA and BLA were punched using 20g blunt syringe needle (Warner Instruments). All tissues were immediately frozen on dry ice and kept at -80 °C until RNA extraction.

*Locus coeruleus, central amygdala and basolateral amygdala.* Total mRNA was extracted from tissue using the Arcturus PicoPure RNA Isolation Kit (Thermo Fisher Scientific, Waltham, MA). Around 1 mg of LC tissue was incubated for 30 min in 50 µl of extraction buffer at 42 °C, (500 rpm) and then centrifuged for 2 min at 3,000 g. The supernatant (50 µl) was transferred in a new tube containing 50 µl of 70% EtOH. The mix was then loaded on an RNA Purification Column (pre-conditioned with 250 µl of conditioning buffer for 5 min), centrifuged at 100 g for 2 min (binding of the RNA to the column), and at 16,000 g for 30 s to remove the flowthrough. Next 100 µl of washing buffer 1 (WB1) was added and centrifuged at 8,000 g, for 1min, before adding 10 µl of DNAse and 30 µl of RDD buffer (Qiagen, Germany). The mix was left at room temperature for 15 min before adding 40 µl of WB1 and centrifuge at 8,000 g for 15 s. The column was then washed two times by adding 100 µl of washing buffer 2, centrifuged at 8.000 g for 1 min after the first wash, and two times at 16.000 g for 1 min after the second wash to remove all traces of buffer. Columns were transferred to a new 0.5 ml collection tube, to which 12 µl of elution buffer was added, left at room temperature for 1 min, and centrifuged at 1.000 g for 1 min to distribute the elution buffer on the column. Finally, the RNA was eluted by centrifugation for 1 min at 16.000 g. RNA concentrations were measured by spectrophotometry (Nanodrop One, Thermo Fisher Scientific, Waltham, MA). Samples were then kept at −80 °C until use.

*Anterior cingulate cortex* . Total mRNA was extracted from tissue using the Qiagen RNeasy minikit (Qiagen, Germany). Around 20mg of tissue were transferred to bead lysis 1.5 mL tubes (Pink RINO RNA lysis kit, Next Advance, Troy, NY) filled with 700 µl of TRIzol^TM^ (Thermo Fisher Scientific, Waltham, MA) and spun in a bullet blender (Next Advance, Troy, NY) for 5 min at full speed. The lysate was retrieved, transferred to a new tube, and left for 5 min at room temperature. It was then mixed with 140 µl of chloroform, left for 3 min at room temperature, and centrifuge at 12.000 g for 15 minutes, at 4 °C. Next, the supernatant was transferred to a new tube and mixed with 1.5 volume of 100% ethanol. The mix was loaded on an RNA Purification Column and centrifuged at 8.000 g for 15 s at room temperature. Then, 10 µl of DNAse and 30 µl of RDD buffer (Qiagen, Germany) were added on the column and at room temperature for 15 min. The column was then washed one time with 700 µl of RWT buffer (8.000, 15s, room temperature), 2 times with 500 µl of RPE buffer (8.000, 15s, room temperature) and spined another time for 1 min at full speed to dry the membrane. The column was placed in a new 1.5 mL collection tube and 30 of RNase-free water was loaded on the membrane. Finally, the RNA was eluted by centrifugation for 1 min at 8.000 g. RNA concentrations were measured by spectrophotometry (Nanodrop One, Thermo Fisher Scientific, Waltham, MA). Samples were then kept at −80 °C until use.

RT-qPCR

Tissue was prepared as for electrophysiology and tissue punches were made to isolate individual brain regions as described previously[43]. To generate cDNA, 50 ng of the total mRNA was reversed transcribed with a qScript cDNA synthesis kit (QuantaBio, Beverly, Massachusetts) following manufacturer’s instructions. Real-time quantitative polymerase chain reaction (RT-qPCR) was performed in 10 μL reaction containing 2 μL of cDNA (1/10 dilution), 5 μL of PowerUp SYBR Green Master Mix (Applied Biosystems, Foster City, California, United-States), 2 μL of a mix of forward and reverse primers (10 μM) and 1 μL of H2O. The cycling conditions were 50 °C for 2 min, 95 °C for 10 min, and then 40 cycles at 95 °C for 15 s and 60 °C for 1 min. The following primers were used:

*B2m*: F: TGCTACGTAACACAGTTCCACC; R: TCTGCAGGCGTATGTATCAGTC

*Dbh*: F: CCGAAATGCCAAAATTGTCA; R: GGACCCCTGCCTGTATTTTGT

*Th*: F: TGCAGCCCTACCAAGATCAAAC ; R: CGCTGGATACGAGAGGCATAGTT

*Adra1a*: F: TGCAGCCCTACCAAGATCAAAC ; R: CGCTGGATACGAGAGGCATAGTT

*Adra2a*: F: TGCAGCCCTACCAAGATCAAAC ; R: CGCTGGATACGAGAGGCATAGTT

*Adrb2*: F: TGCAGCCCTACCAAGATCAAAC ; R: CGCTGGATACGAGAGGCATAGTT

*Crhr1*: F: GGAACCTCATCTCGGCTTTCA ; R: GTTACGTGGAAGTAGTTGTAGGC

Data were normalized to *B2m* (from the same animal), and fold changes were calculated using the 2^-ΔΔCt^ method [44].

Immunohistochemistry

Mice were anesthetized with an i.p. injection of a cocktail containing ketamine (69.57 mg/ml), xylazine (4.35 mg/ml), & acepromazine (0.87 mg/ml). They were then perfused with 20 ml of cold 1 x PBS and then 20 ml of cold 4% paraformaldehyde in 0.1M PB. Brains were dissected and postfixed in paraformaldehyde for 24 hours at 4°C. After, the brains were left in 30% sucrose in 0.05M PB for 72 hours. Next, the brains were frozen and sliced into 30 μm thick sections using a microtome (SM2000R, Leica, Germany). Sections were rinsed three times with PBS and then incubated in a blocking solution containing 2% bovine serum albumin (BSA) plus 5% normal goat serum (NGS) in PBST for one hour before transferring to a PBS solution containing c-Fos (9F6) (rabbit, 1:1000, Cell Signaling Technology, 2250) and tyrosine hydroxylase (TH) (#TYH, 1:1000; Aves Labs Inc., Tigard, OR, USA) primary antibodies overnight on a shaker at room temperature. Sections were then washed in PBS 3 times and incubated in PBS with secondary antibodies Alexa Flour 488 anti-rabbit (1:400; Cat#A-11008; Invitrogen, Carlsbad, California, USA) and Alexa Flour 594 anti-chicken (1:1000; Cat#A-11042; Invitrogen, Carlsbad, California, USA) for 2 hours at room temperature followed by a rinse with PBS for three times. Sections were then mounted on glass slides with Vectashield mounting medium (Vector Labs, CA, USA). Images were collected using a Leica confocal microscope (SP8, Leica, Germany). cFos, TH, and DAPI staining were analyzed using ImageJ. To measure both cFos and DAPI, a region-of-interest (ROI) was created around the LC and copied onto the channel that had the staining. A threshold for the cFos or DAPI intensity was then created. A binary image of the cFos or DAPI ROI was then made to isolate overlapping signals and count the staining only located within the ROI (**Figure S3**). ImageJ was used to count the number of TH-positive cells within an image of the LC (no ROI was used). The intensity feature of ImageJ was also used to measure the intensity of the TH signal.

Stereotaxic Surgery

Mice were anesthetized in an induction chamber (3% isoflurane) and placed in a stereotaxic frame (Kopf Instruments, Model 940) where they were maintained at 2-2.5% isoflurane throughout the procedure. Preoperative analgesia with carprofen (5 mg/kg, s.c.) and intraoperative bupivicaine (0.25%, intradermal) and lidocaine (0.05 mg/kg, topical) was used.A craniotomy was performed, and mice were injected with 350 nL of AAV8-hSyn-DIO-hM4D(Gi)-mCherry (Addgene viral prep # 44362-AAV8) or AAV8-hSyn-DIO-mCherry (Addgene viral prep # 50459-AAV8) bilaterally into the locus coeruleus. Both viruses were gifts from Bryan Roth (Stereotaxic coordinates from bregma: -5.45 mm anterior-posterior (AP), ± 1.10 mm medial-lateral (ML), and -3.75 mm dorsal-ventral (DV) were used. Postoperative care included triple antibiotic ointment (topical) and *ad lib* access to carprofen tablets (a total of ¼ tablet per mouse (2 mg carprofen per 5 g tablet, available for three days) on the floor of their home cage, and a 0.15 mL subcutaneous saline injection immediately following surgery. Mice were allowed to recover for at least four weeks prior to behavioral testing and slice electrophysiology.


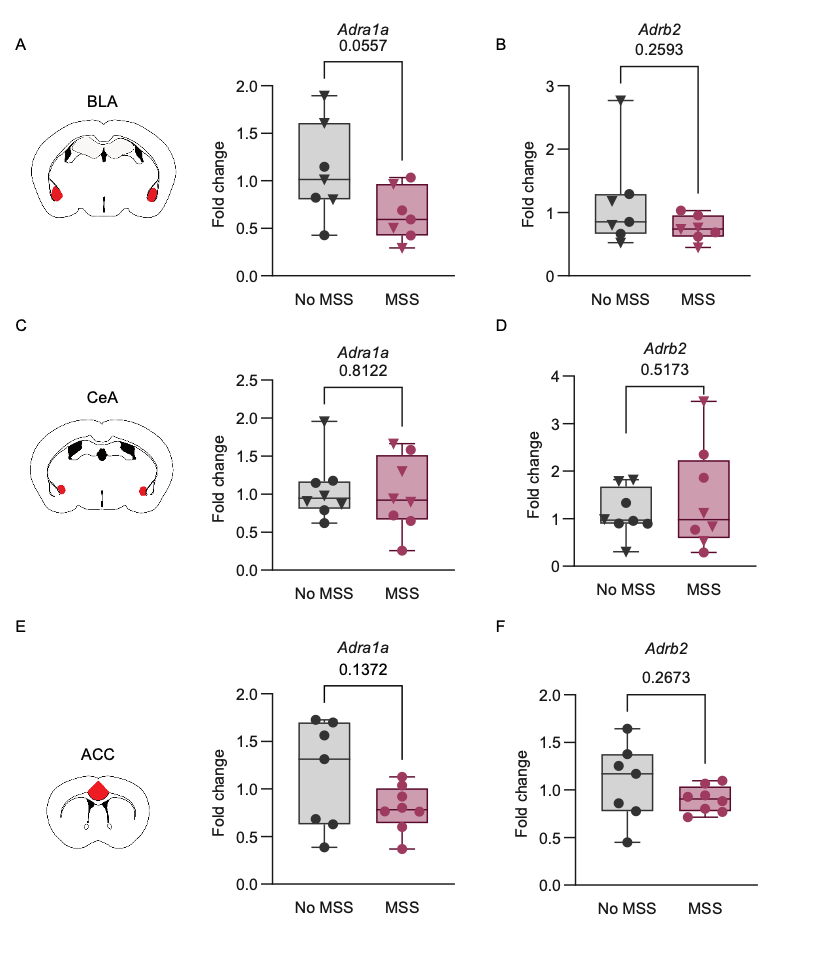
**Supplementary Figure 1: mRNA Expression in LC projection areas in MSS and No MSS mice.** (**A**) In the BLA, *Adra1a* expression displayed a downward trend in MSS animals compared to animals with No MSS (unpaired t-test, t(12) = 2.118, p= 0.0557). (**B**) *Adrb2* expression in the BLA was comparable between MSS animals and No MSS animals (Mann-Whitney test, U=15, p= 0.2593). (**C**) In the CeA, *Adra1a* expression was comparable between MSS animals and no MSS (unpaired t-test, t(14) = 0.2421, p= 0.8122). (**D**) *Adrb2* expression in the CeA was comparable between MSS animals and No MSS animals (unpaired t-test, t(14) = 0.6644, p= 0.5173). (**E**) In the ACC, *Adra1a* expression was comparable between MSS animals and No MSS animals (unpaired t-test, t(13) = 1.584, p= 0.1372). (**F**) *Adrb2* expression in the ACC was comparable between MSS animals and No MSS animals (unpaired t-test, t(13) = 1.159, p=0.2673). Data are presented as box and whiskers plots, where the box represents the IQR, the line within the box indicates the median, and whiskers extend from the minimum to maximum values.

**
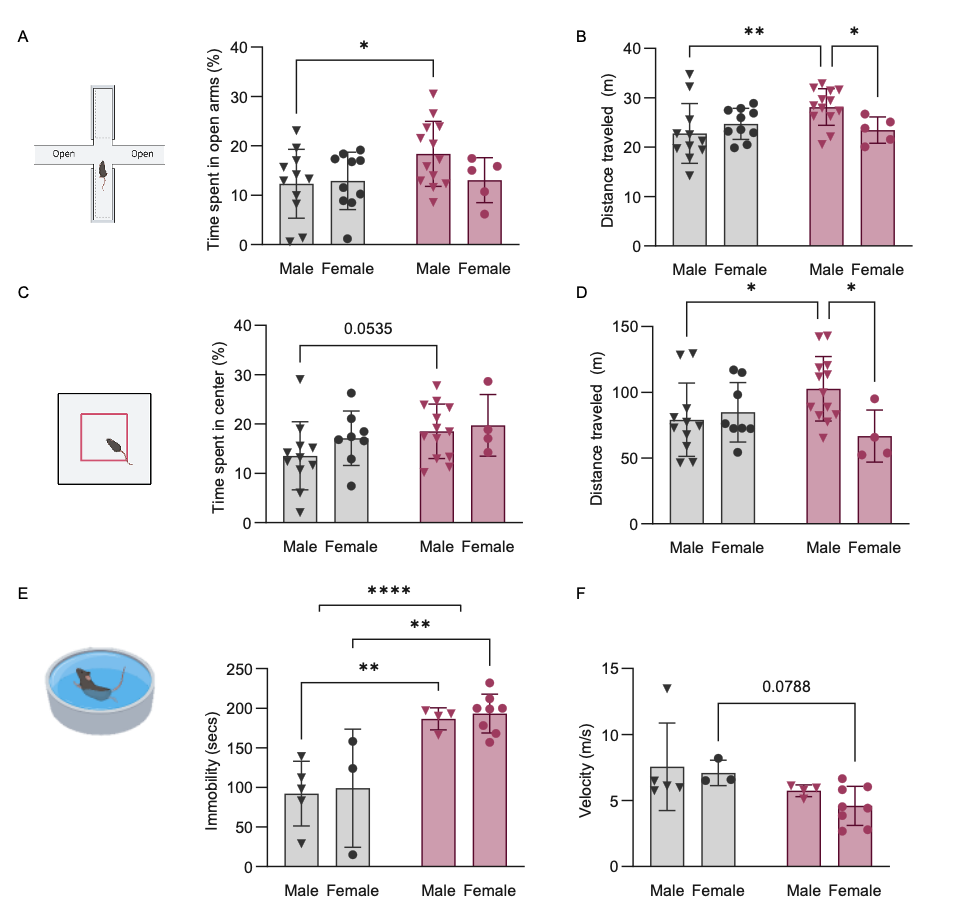
**

**Supplementary Figure 2: EPM, OFT, and FST data not separated by sex.** (**A**) The percent of time spent in the open arms of the elevated plus maze (Two-way ANOVA with Uncorrected Fisher's LSD, F(1, 37) = 1.739, p= 0.0337). (**B**) The total amount of distance moved in the elevated plus maze (Two-way ANOVA with Uncorrected Fisher's LSD, F(1, 35) = 5.048, **p= 0.0045 and *p= 0.047). (**C**) The percent of time spent in the center of the open field test (Two-way ANOVA with Uncorrected Fisher's LSD, F(1, 32) = 2.881). (**D**) The total distance moved in the open field test (Two-way ANOVA with Uncorrected Fisher's LSD, F(1, 30) = 10.78, ***p=0.0008 and **p= 0.0063). (**E**) The amount of time spent immobile during the forced swim test for the No MSS and MSS group (Two-way ANOVA with Uncorrected Fisher's LSD, F(1, 16) = 27.70,****p= <0.0001, **p= 0.0018 and **p=0.0019, respectively). (**F)** The average velocity during the forced swim test for the No MSS and MSS group (Two-way ANOVA with Uncorrected Fisher's LSD, F(1, 16) = 5.314). Triangles ▼ in graphs indicate male animals; circles ● indicate female animals. All data represented as mean ± SEM.

**
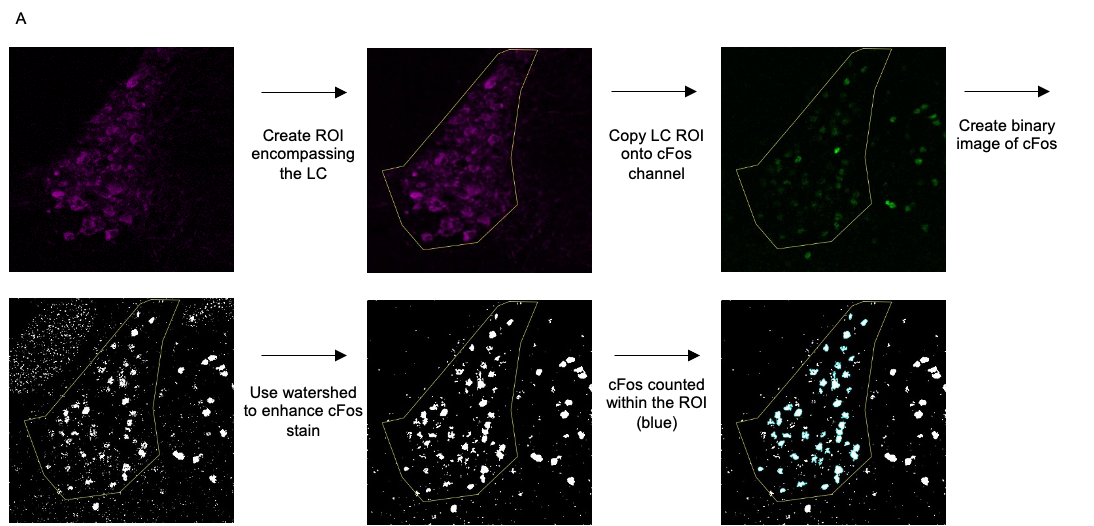
**

**Supplementary Figure 3: Using ImageJ to analyze cFos in the LC region.** Schematic of the process to analyze cFos after immunohistochemistry staining as described in the methods.
